# Family Engagement in Cardiac Intensive Care and Patient Outcomes: Insights From the NGAGE Trial

**DOI:** 10.1016/j.jacadv.2025.101946

**Published:** 2025-10-01

**Authors:** Joshua Kandiah, Michael Goldfarb

**Affiliations:** a Department of Cardiology, McGill University, Montreal, Quebec, Canada; b Division of Cardiology, Jewish General Hospital, McGill University, Montreal, Quebec, Canada

**Keywords:** cardiac ICU, family engagement, patient outcomes

**Figure:**
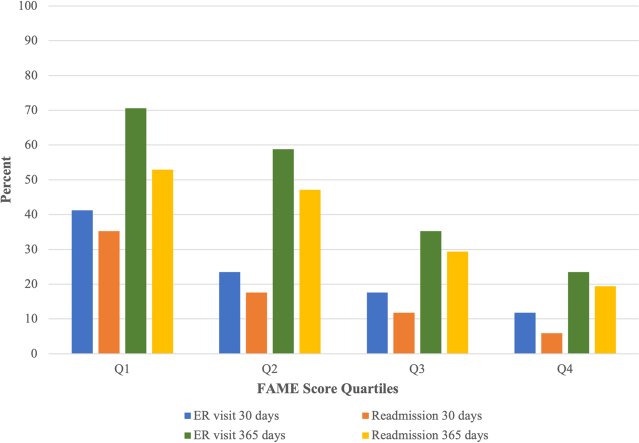

Family members are increasingly recognized as important contributors to patient care in the cardiac intensive care unit (ICU).^1^ While a growing evidence base supports family engagement in patient care in the cardiac ICU to improve family experience and satisfaction, little is known about the relationship between family involvement and patient outcomes. Insufficient evidence for patient benefit from family involvement is a critical knowledge gap that has impaired efforts for implementation of family engagement practices in the cardiac ICU.^1^ Thus, the objective of this study was to explore the relationship between family involvement in the cardiac ICU and patient outcomes using data from the NGAGE trial.

**What is the clinical question being addressed?**

What is the relationship between family involvement in the cardiac ICU and patient outcomes?

**What is the main finding?**

Higher levels of family engagement in care were associated with reduced rates of ED visits and hospital readmission.

The NGAGE trial was a prospective pilot randomized controlled trial of 88 family members of cardiac ICU and ward patients at an academic tertiary care center in Montreal, Canada, from July 2023 to April 2024.^2^ The current study is a retrospective review of patient data from all family members enrolled in the NGAGE trial. Institutional ethics approval was obtained for this study (CIUSSS West-Central Montreal).

The NGAGE trial involved family members being randomized 1:1 to the intervention (use of the NGAGE tool) or usual care. “Family” was considered anyone with a biological, legal, or emotional relationship with the patient, and with whom the patient wished to have involved in their care. Family participants in the intervention group were given access to NGAGE, a digital communication tool designed to empower family members to actively participate in their loved one’s care. Participants using the NGAGE web-based tool have the option to engage or report. Engage allows family members to request a specific engagement activity (eg, communicate with the health care team, receive information or education, participate in decision-making, or perform direct care activities such as mobilization). The appropriate health care team member is then informed about the engagement request. Report allows family members to respond to study questionnaires. The usual care group followed the hospital’s standard policies for family engagement in care.

The following outcome data were collected from family members: engagement (FAMily Engagement [FAME]), mental health (Hospital Anxiety and Depression Scale), and satisfaction (Family Satisfaction-ICU). The following patient data were gathered from the electronic record: age, sex, admission diagnosis, hospital length of stay, discharge destination, and emergency department (ED) visit or readmission within 30 or 365 days of index admission. The outcomes of interest were the relationship between the family engagement (FAME) score and patient outcomes (length of stay, discharge destination, ED visits, and readmission). FAME scores were grouped by quartiles and the highest quartile (Q4; most engaged in care) was compared to the lowest quartile (Q1; least engaged in care). Between-group differences were compared using the *t*-test for continuous variables and the chi-squared test for categorical variables. Logistic regression was used to determine the association between FAME and covariates of interest with ED visits at 30 days and 365 days. A *P* value of ≤0.05 was considered statistically significant.

There were 88 patients (age 74.6 ± 13.5 years; 35% female) included in the analysis. There were no baseline between-group differences (lowest vs highest FAME quartile) in patient demographics (age, sex) or primary admission diagnoses (all *P* > 0.05). The most common admission diagnoses were acute coronary syndrome (32%), coronary artery bypass graft (22%), and heart failure (19%). Twelve patients (14%) received mechanical ventilation and sedation during hospitalization.

Patients with family members in the highest FAME quartile had a lower rate of ED visits at 30 days (11.8% vs 41.2%; *P* = 0.050) and 365 days (23.5% vs 70.6%; *P* = 0.005) and hospital readmissions at 30 days (5.9% vs 35.3%; *P* = 0.03) as compared to patients with family members in the lowest FAME quartile. There were no differences in length of hospital stay or discharge destination (both *P* > 0.05). Trends in ED visits and readmission rates across FAME quartiles are shown in Figure 1. The FAME score, but not patient age, sex, or hospital length of stay, was a predictor of ED visits at 30 and 365 days in the regression analysis.

**Figure 1:**
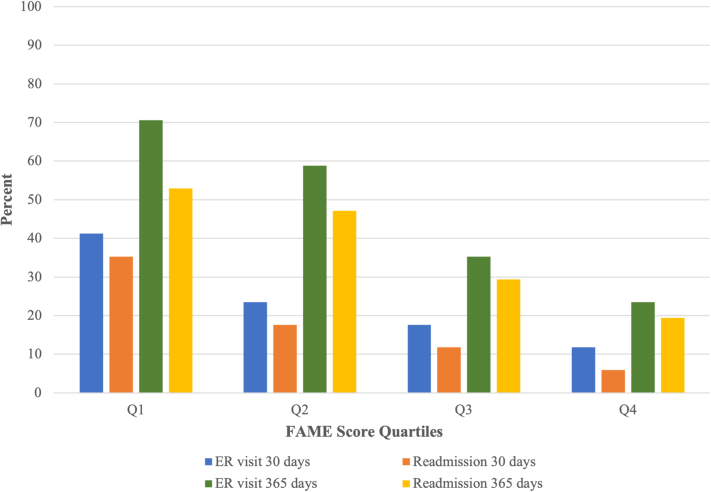
Emergency Department Visits and Hospital Readmission Across FAME Quartiles ER = emergency room; FAME = Family Engagement.

Patients of NGAGE tool users had lower ED visits at 30 days as compared to patients of the usual care group (15.8% vs 32.3%; *P* = 0.04). Higher Family Satisfaction-ICU scores were moderately correlated with lower rates of ED visits and hospital readmissions at 30 and 365 days (range r = −0.22 to 0.28; *P* < 0.05). There was no correlation between Hospital Anxiety and Depression Scale scores and ED visits or hospital readmissions (*P* > 0.05).

The key finding in our study was that higher levels of family engagement in care were associated with reduced rates of ED visits and hospital readmission. Family satisfaction was also associated with reduced rates of ED visits and hospital readmission. These findings support the need for prospective studies to explore whether strategies that increase family involvement in care can improve patient outcomes in the cardiac ICU.

The mechanism by which family engagement in patient care during hospitalization improves posthospital outcomes is not clear. Family members who are more involved in care during hospitalization may provide more support to the patient after they leave hospital as well, since family members often serve as informal, unpaid caregivers of cardiac ICU survivors postdischarge. Family engagement in patient care may also result in a better understanding of the patients’ condition leading to better postdischarge adherence with treatment plans and follow-up care.

As this was a retrospective study of available administrative data, no patient-reported outcomes were included. Furthermore, measures of patient acuity (ie, critical care scores) were not available in our database. Patient acuity and complexity may influence the dynamics of family engagement and should be explored in future studies. There is also a need to prospectively collect patient-reported outcomes and experience measures to better gauge the impact of family involvement on the cardiac ICU patient.

In conclusion, increased family engagement in patient care was possibly associated with improved posthospital patient outcomes. Further research is needed to explore whether interventions that increase family engagement in care can improve patient outcomes during and after cardiac ICU stay.

## Funding support and author disclosures

Dr Goldfarb is supported by a Clinical Research Award from the Fonds de recherche du Quebec Sante. The NGAGE trial received operating grant support from the McGill Nursing Collaborative for Education and Innovation in Patient- and Family-Centered Care (Newton Foundation/10.13039/501100020986Jewish General Hospital Foundation). Dr Kandiah has reported that he has no relationships relevant to the contents of this paper to disclose.
